# A prospective cohort study of periostin as a serum biomarker in patients with idiopathic pulmonary fibrosis treated with nintedanib

**DOI:** 10.1038/s41598-023-49180-4

**Published:** 2023-12-27

**Authors:** Masaki Okamoto, Kiminori Fujimoto, Takeshi Johkoh, Atsushi Kawaguchi, Hiroshi Mukae, Noriho Sakamoto, Takashi Ogura, Satoshi Ikeda, Yasuhiro Kondoh, Yasuhiko Yamano, Kosaku Komiya, Kenji Umeki, Hirotaka Nishikiori, Yoshinori Tanino, Toru Tsuda, Naoki Arai, Masamichi Komatsu, Susumu Sakamoto, Kazuhiro Yatera, Yoshikazu Inoue, Yasunari Miyazaki, Seishu Hashimoto, Yasuo Shimizu, Hironao Hozumi, Hiroshi Ohnishi, Tomohiro Handa, Noboru Hattori, Tomoo Kishaba, Motoyasu Kato, Minoru Inomata, Hiroshi Ishii, Naoki Hamada, Satoshi Konno, Yoshiaki Zaizen, Arata Azuma, Takafumi Suda, Kenji Izuhara, Tomoaki Hoshino

**Affiliations:** 1 Division of Respirology, Neurology, and Rheumatology, Department of Internal Medicine, Kurume University School of Medicine, 67 Asahi-machi, Kurume, Fukuoka 830-0011 Japan; 2 Department of Respirology, NHO Kyushu Medical Center, 1-8-1 Jigyohama, Chuo-ku, Fukuoka, 810-0065 Japan; 3 Department of Radiology, Kurume University School of Medicine, 67 Asahi-machi, Kurume, Fukuoka 830-0011 Japan; 4 Department of Radiology, Kansai Rosai Hospital, Inabasou 3-1-69, Amagasaki, Hyogo 660-0064 Japan; 5 Education and Research Center for Community Medicine, Faculty of Medicine, Saga Medical School, 5-1-1 Nabeshima, Saga, 849-8501 Japan; 6 Department of Respiratory Medicine, Nagasaki University Graduate School of Biomedical Sciences, 1-7-1 Sakamoto, Nagasaki, 852-8501 Japan; 7 Division of Respiratory Medicine, Kanagawa Cardiovascular and Respiratory Center, 6-16-1 Tomiokahigashi, Yokohama, Kanagawa-ku, Kanagawa 236-0051 Japan; 8 Department of Respiratory Medicine and Allergy, Tosei General Hospital, 160 Nishioiwake, Seto, Aichi 489-0065 Japan; 9 Respiratory Medicine and Infectious Diseases, Faculty of Medicine, Oita University, 1-1 Idaigaoka, Hasama-machi, Yufu, Oita 879-5593 Japan; 10 Department of Respiratory Medicine, Tenshindo Hetsugi Hospital, 5956 Nakahetsugi, Oita, 879-7761 Japan; 11 Department of Respiratory Medicine and Allergology, Sapporo Medical University School of Medicine, South-1-West-16, Chuo-ku, Sapporo, Hokkaido 060-8543 Japan; 12 Department of Pulmonary Medicine, School of Medicine, Fukushima Medical University, 1 Hikarigaoka, Fukushima, Fukushima 960-1295 Japan; 13 Kirigaoka Tsuda Hospital, 3-9-20 Kirigaoka, Kitakyushu, Fukuoka 802-0052 Japan; 14 Department of Respiratory Medicine, National Hospital Organization Ibarakihigashi National Hospital, 825 Terunuma, Tokai-mura, Ibaraki 319-1113 Japan; 15 First Department of Internal Medicine, Shinshu University School of Medicine, 3-1-1 Asahi, Matsumoto, Nagano 390-8621 Japan; 16 Department of Respiratory Medicine, Toho University Omori Medical Center, 6-11-1 Omorinishi, Tokyo, 143-8541 Japan; 17 Department of Respiratory Medicine, University of Occupational and Environmental Health, 1-1 Iseigaoka, Kitakyushu, Fukuoka 807-8555 Japan; 18 Clinical Research Center, National Hospital Organization Kinki-Chuo Chest Medical Center, 1180 Nagasone-cho, Sakai, Osaka 591-8555 Japan; 19 Department of Respiratory Medicine, Graduate School of Medical and Dental Sciences, Tokyo Medical and Dental University, 1-5-45 Yushima, Tokyo, 113-8510 Japan; 20 Department of Respiratory Medicine, Tenri Hospital, 200 Mishima-cho, Tenri, Nara 632-8552 Japan; 21 Department of Pulmonary Medicine and Clinical Immunology, Dokkyo Medical University School of Medicine, 880 Kitakobayashi, Mibu, Shimotsuga, Tochigi 321-0293 Japan; 22 Second Division, Department of Internal Medicine, Hamamatsu University School of Medicine, 1-20-1 Handayama, Hamamatsu, Shizuoka 431-3192 Japan; 23 Department of Respiratory Medicine and Allergology, Kochi Medical School, Kochi University, 185-1 Kohasu, Nankoku, Kochi 783-8505 Japan; 24 Department of Advanced Medicine for Respiratory Failure, Graduate School of Medicine, Kyoto University, 54 Shogoin Kawaharacho, Sakyo-ku, Kyoto 606-8507 Japan; 25 Department of Molecular and Internal Medicine, Graduate School of Biomedical Sciences, Hiroshima University, 1-2-3 Kasumi, Minami-ku, Hiroshima 734-8551 Japan; 26 Department of Respiratory Medicine, Okinawa Chubu Hospital, 281 Miyazato, Uruma, Okinawa 904-2293 Japan; 27 Department of Respiratory Medicine, Juntendo University Graduate School of Medicine, 2-1-1 Hongo, Tokyo, 113-8421 Japan; 28 Department of Respiratory Medicine, Japanese Red Cross Medical Center, 4-1-22 Hiroo, Tokyo, 150-8935 Japan; 29 Department of Respiratory Medicine, Fukuoka University Chikushi Hospital, 1-1-1 Zokumyouin, Chikushino, Fukuoka 818-8502 Japan; 30 Department of Respiratory Medicine, Fukuoka University Hospital, 7-45-1 Nanakuma, Fukuoka, 814-0180 Japan; 31 Department of Respiratory Medicine, Faculty of Medicine, Hokkaido University, N15W7 Kita-ku, Sapporo, Hokkaido 060-8638 Japan; 32 Respirology and Clinical Research Center, Mihara General Hospital and Nippon Medical School, Tokorozawa, Saitama 359-0045 Japan; 33 Division of Medical Biochemistry, Department of Biomolecular Sciences, Saga Medical School, 5-1-1 Nabeshima, Saga, 849-8501 Japan

**Keywords:** Biomarkers, Medical research

## Abstract

This study investigated the utility of periostin, a matricellular protein, as a prognostic biomarker in patients with idiopathic pulmonary fibrosis (IPF) who received nintedanib. Monomeric and total periostin levels were measured by enzyme-linked immunosorbent assay in 87 eligible patients who participated in a multicenter prospective study. Forty-three antifibrotic drug-naive patients with IPF described in previous studies were set as historical controls. Monomeric and total periostin levels were not significantly associated with the change in forced vital capacity (FVC) or diffusing capacity of the lungs for carbon monoxide (D_LCO_) during any follow-up period. Higher monomeric and total periostin levels were independent risk factors for overall survival in the Cox proportional hazard model. In the analysis of nintedanib effectiveness, higher binarized monomeric periostin levels were associated with more favorable suppressive effects on decreased vital capacity (VC) and D_LCO_ in the treatment group compared with historical controls. Higher binarized levels of total periostin were associated with more favorable suppressive effects on decreased D_LCO_ but not VC. In conclusion, higher periostin levels were independently associated with survival and better therapeutic effectiveness in patients with IPF treated with nintedanib. Periostin assessments may contribute to determining therapeutic strategies for patients with IPF.

## Introduction

Idiopathic pulmonary fibrosis (IPF), pathologically usual interstitial pneumonia (UIP), is the most common idiopathic interstitial pneumonia (IIP) with unknown etiology^1–3^. Patients with IPF have a grave prognosis, with a median survival of approximately 3 years^1–4^. The decrease in forced vital capacity (FVC) or diffusing capacity of the lung for carbon monoxide (D_LCO_) and acute exacerbation have been reported as significant risk factors for mortality in IPF patients^3–8^. Recently, two fibrotic drugs for IPF, nintedanib and pirfenidone, have been shown for safety and efficacy^9,10^. Treatment with nintedanib reduced the decrease in FVC, the incidence of acute exacerbation of IPF, and the worsening health-related quality of life in two replicate randomized, double-blind, phase 3 trials (INPULSIS-1 and INPULSIS-2)^9^. Moreover, a meta-analysis of 12,956 patients with IPF across 8 randomized controlled trials (RCT) and 18 cohort studies showed that treatment with antifibrotic drugs including nintedanib and pirfenidone reduced the risk of all-cause mortality and acute exacerbation^11^. Therefore, nintedanib and pirfenidone therapy for IPF is recommended in the global and Japanese guidelines^1,2,12^. However, determining the appropriate timing of therapy initiation for IPF is difficult because the rate of disease progression varies among individuals^13^. Thus, a prognostic factor is required for the decision-making of therapeutic strategies for IPF^13^. Nevertheless, there is no established biomarker to predict mortality, progression, or therapeutic response in IPF patients. In previous reports, the gender-age-pulmonary physiology (GAP) stage based on clinical, e.g., gender or age, and physiological, e.g., FVC and D_LCO_, variables can predict overall survival in IPF patients^14,15^. Moreover, some blood biomarkers such as monocyte count, circulating protein fragments degraded by matrix metalloproteases (MMPs) MMP1 and MMP7, C–C motif chemokine ligand 18 (CCL18), Krebs von den Lungen-6 (KL-6), surfactant protein D (SP-D), and surfactant protein A (SP-A) were reported to be associated with decreased pulmonary function, incidence of acute exacerbation, and/or mortality in IPF patients^16–24^.

Periostin is an extracellular matrix and matricellular protein that modulates cell–matrix interactions via αvβ1, αvβ3, or αvβ5 integrin receptor^25^. Periostin is secreted from fibroblasts, epithelial cells, and endothelial cells via stimulation by interleukin (IL)-4, IL-13, and transforming growth factor (TGF)-β, and contributes to the pathophysiology of fibrosis during systemic sclerosis, allergic rhinitis, chronic rhinosinusitis, and atopic dermatitis^26–28^. In addition, we previously reported upregulation of periostin in the lung tissue of mice with bleomycin-induced lung injury and its increase expression in the lungs and serum of human IIP^29,30^. We and other groups have reported that higher serum periostin levels were associated with decreased pulmonary function and shortened overall survival and time-to-event, including more than a 5% decrease in FVC, acute exacerbation or death, and increases in the extent of abnormal findings on high-resolution computed tomography (HRCT) in patients with IPF^30–32^. Unfortunately, periostin is not a specific biomarker for IPF because it is upregulated in various other diseases^26–28^. Izuhara et al. established a new enzyme-linked immunosorbent assay (ELISA) kit that specifically detects the monomeric form (SS20A × SS19D, capture and detection antibody). The level of monomeric periostin is more specific for IPF compared with that measured by conventional ELISA kits that detect both the monomeric and oligomeric forms (SS18A × SS17B, total periostin)^33^. We showed that both serum monomeric and total periostin were associated with decreases in FVC and D_LCO_ in a multicenter prospective analysis^33^. Moreover, serum periostin was reported to be associated with mortality in patients with acute exacerbation of fibrotic interstitial lung disease (ILD) and fibrotic hypersensitivity pneumonia^34,35^.

However, the patients who participated our previous studies included many antifibrotic drug therapy-naive individuals. The specific objectives in the present study were clarifying the association of monomeric and total periostin levels with decreased pulmonary function and survival in IPF patients treated with nintedanib and nintedanib effectiveness.

## Patients and methods

### Study participants

The present study was a multicenter prospective cohort study. Study samples were obtained from consecutive patients with IPF who received care at 19 collaborative facilities from 2015 to 2021. The main inclusion criteria for participation were as follows: patients diagnosed with IPF based on global guidelines and scheduled to start nintedanib therapy within 2 months after enrollment; and aged ≥ 40 years. Similarly, the main exclusion criteria were as follows: patients who developed acute exacerbation within 3 months prior to enrollment; pregnancy; corticosteroid therapy of more than 10 mg per day of prednisolone equivalent; immunosuppressants; and pirfenidone, N-acetylcysteine, and any investigational treatments for IPF. Patients who could not continue nintedanib therapy or be observed for more than 3 months from baseline were dropped out and the data of these patients were excluded from analyses. Eligible patients with IPF were selected according to multidisciplinary discussion diagnosis following global criteria^3^ by two each of board-certified clinical, radiological investigators who had considerable experience in thoracic diagnosis. Histological diagnoses of cases that underwent lung biopsies were performed individually at collaborating facilities. Diagnosis of acute exacerbation of IPF was defined in accordance with the criteria detailed in a previous report^36^. As historical control data for evaluating nintedanib effectiveness, we used serum biomarker levels measured in 43 antifibrotic drug-naive patients with IPF who were selected from 60 patients who participated in the CoDD-PF or Kurume study^30,33^.

### Study protocol

The date of serum collection before the start of nintedanib therapy was set as the baseline (day 0). Sera were collected for measuring the level of periostin every 6 months from baseline. We evaluated pulmonary function, serum level of lactate dehydrogenase (LDH), KL-6, and SP-D as well as the modified Medical Research Council (mMRC) dyspnea scale every 6 months for up to 12 months after baseline. Similarly, chronic obstructive pulmonary disease assessment test (CAT), distance and minimum SpO_2_ on a 6-min walk test at baseline and 12 months later, was performed. Chest HRCT examinations without contrast medium were performed at baseline using a variety of scanners. Beyond 1 year after baseline, only outcomes including death and acute exacerbation were observed up to completion of the study.

The treatment protocol with nintedanib was based on recommendations similar to those provided in the INPULSIS trials^9^. The recommended starting dose of nintedanib is 150 mg twice a day. Dose reductions of up to 100 mg twice a day and treatment interruptions are recommended to manage adverse events. After improvement of adverse events, nintedanib can be reintroduced upon the decision of the investigator.

The protocol of HRCT and diagnosis with radiological pattern based on global guidelines were performed as reported previously^3,30,32,33^. Two board-certificated radiologists with 35 and 33 years of experience, respectively, who specialized in diffuse lung diseases with experience in chest CT interpretation, independently evaluated the HRCT findings. After assessing the interobserver agreement, the final decision was made by consensus. The radiologists were blinded to clinical information.

### Measurement of periostin by ELISA

Duplicated serum samples were obtained from study participants and then stored at − 80 °C until human periostin ELISA assay, which we previously established^33^.

### Statistical analysis

Data are expressed as the median (25th–75th percentiles of the interquartile range) or least squares mean ± standard error. Differences between two groups were analyzed as appropriate using the Wilcoxon rank-sum test or Fisher’s exact test. Associations between two groups were analyzed using the Spearman’s rank correlation coefficient. The agreement between two independent observers was assessed using Cohen’s kappa statistics when classifying HRCT patterns according to the IPF guidelines. Progression of IPF was defined as a greater than 5% decrease in the FVC over 6 or 12 months based on a previous report^6^. Using the Kaplan–Meier method, the overall survival was compared by log-rank test and cut-off levels were set based on previous studies as follows: D_LCO_ at baseline, 39%; monomeric and periostin level, 15 ng/mL and 100 ng/mL, respectively; and decrease in FVC and D_LCO_ over 6 or 12 months, 5% and 15%, respectively^6–8,33^. In Cox proportional hazards model analysis, we detected variables significantly (*P* < 0.05) associated with overall survival by univariate analyses. Cox analysis adjusted for age, gender, FVC, and D_LCO_ at baseline as confounding factors was performed to assess whether baseline changes in serum monomeric and total periostin levels were associated with overall survival by multivariate analysis. For evaluating the association between biomarker level and nintedanib effectiveness, relative changes in VC and D_LCO_ during the 6-month period after baseline were compared between the study participants (treatment group) and historical controls according to biomarker-high and -low groups. The median levels of each biomarker in the study participants were set as the cut-off levels. Data of the treatment group and historical controls were compared by multiple regression analysis adjusted for age, VC, and D_LCO_ at baseline. *P* < 0.05 was taken to represent statistical significance. All statistical analyses were performed using R Statistical Software (version R 4.2.0; R Foundation for Statistical Computing, Vienna, Austria) and JMP 14.0 (SAS Institute Japan, Tokyo, Japan).

### Ethical approval and consent to participate

We received specific approval for all procedures from the Institutional Review Board (IRB) of Kurume University School of Medicine and collaborative facility in accordance with the ethical standards of the Helsinki Declaration of 2013. The approval numbers (date of IRB approval) of our study are 08067 (August 2, 2008), 11216 (February 29, 2012), and 15096 (August 11, 2015). Written informed consent was obtained from all patients.

## Results

### Characteristics and outcomes

**Figure 1:**
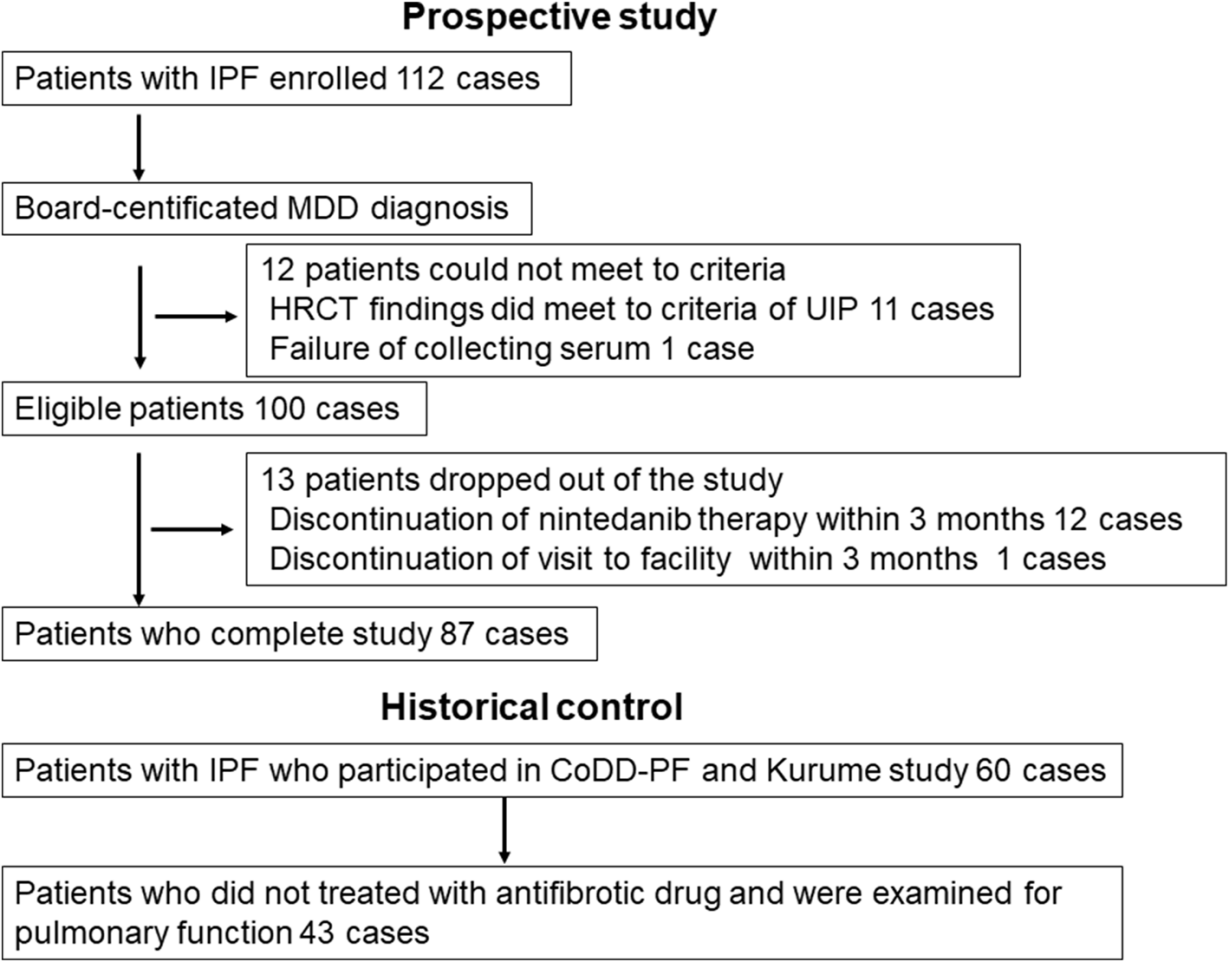
Study enrollment. IPF, idiopathic pulmonary fibrosis; MDD, multidisciplinary discussion; HRCT, high resolution computed tomography.

Study enrollment is shown in Fig. 1. Among 112 enrolled patients with IPF, 12 patients did not meet the study criteria, because of the failure to meet to HRCT criteria of UIP in 11 patients or serum collection in 1 patient. Among 100 eligible patients, 13 patients dropped out of the study because of discontinuation of nintedanib therapy in 12 patients and failure to visit the facility within 3 months after baseline in 1 patient. Eighty-seven patients completed the study, including 78 males and 75 ex-smokers and 1 current smoker, with a median age of 72.0 (68.0–76.0) years. The agreement between two independent observers in classifying HRCT patterns was good (kappa value, 0.687; *P* < 0.001). The patients’ characteristics and outcomes are shown in Table 1. Fifteen of 87 patients (17%) underwent surgical lung biopsy, among which histological classifications included definite and possible UIP pattern in 12 (80%) and 3 (20%) patients, respectively. Monomeric and total periostin levels in collected sera at baseline were 12.2 (9.6–15.9) ng /mL and 83.0 (66.0–106.0) ng /mL, respectively. Serum levels of LDH, KL-6, and SP-D and FVC, VC, and D_LCO_ were 215.0 (192.0–236.0) IU/L, 802.0 (613.0–1160.0) IU/mL, 272.0 (161.0–358.0) ng/mL, 71.7 (60.9–80.1)%, 69.8 (60.7–80.1)%, and 57.1 (48.7–67.9)% at baseline. Among 87 study participants, corticosteroid therapies and long-term oxygen therapies were performed in 6 (7%) and 8 (9%) patients at baseline. During 1174.0 (928.0–1537.0) days of observation, 23 (26%) patients developed acute exacerbation and 36 (41%) died. Nintedanib therapy was discontinued in 30 (35%) of 87 patients during the observation period. The median duration of treatment with nintedanib was 634.0 (371.0–1239.0) days.

**Table 1:** Patients’ characteristics and outcome.

Number	87	Pulmonary function test	
HRCT pattern		FVC (%)	71.7 (60.9–80.1)
Definite UIP	79 (91%)	VC (%)	69.8 (60.7–80.1)
Possible UIP	8 (9%)	D_LCO_ (%)	57.1 (48.7–67.9)
Performing SLB	15 (17%)	Six-minutes walk test	
Definite UIP	12 (80%)	Minimum SpO_2_ (%)	88.0 (84.0–91.0)
Probable UIP	3 (20%)	Distance (m)	440.0 (345.6–498.7)
Age (year)	72.0 (68.0–76.0)	Combination therapy	
Gender (Male)	78 (90%)	Corticosteroids	6 (7%)
Smoker	76 (87%)	Long-term oxygen therapy	8 (9%)
GAP score	4.0 (3.0–5.0)	Observation period (days)	1174.0 (928.0–1537.0)
GAP stage (1/2/3)	31/48/8	Acute exacerbation	23 (26%)
mMRC grade (0/1/2/3/4)	14/40/23/11	Death	36 (41%)
COPD Assessment Test	13.5 (8.3–20.0)	Cause of death	
Serum biomarkers		Chronic respiratory failure	12 (33%)
Monomeric periostin (ng/mL)	12.2 (9.6–15.9)	Acute exacerbation	13 (36%)
Total periostin (ng/mL)	83.0 (66.0–106.0)	Infection	3 (8%)
LDH (IU/L)	215.0 (192.0–236.0)	Heart disease	2 (6%)
KL-6 (IU/mL)	802.0 (613.0–1160.0)	Unknown or others	6 (17%)
SP-D (ng/mL)	272.0 (161.0–358.0)		

Data are expressed as the median (25th to 75th percentiles of the interquartile range [IQR]), unless otherwise stated. HRCT, high-resolution computed tomography; SLB, surgical lung biopsy; UIP, usual interstitial pneumonia; GAP, Gender Age Physiology; mMRC, modified medical research council; COPD, chronic obstructive pulmonary disease; LDH, lactate dehydrogenase; KL-6, Krebs von den Lungen-6; SP-D, surfactant protein D; FVC, forced vital capacity; VC, vital capacity; D_LCO_, diffusing capacity of the lung for carbon monoxide; IPF, idiopathic pulmonary fibrosis.

### Correlations between clinical parameters and changes in pulmonary function

We analyzed whether serum periostin levels were associated with changes in pulmonary function (Table 2). None of the analyzed biomarkers comprising monomeric, total periostin, LDH, KL-6, or SP-D at baseline were significantly associated with relative changes in FVC or D_LCO_ at any follow-up period (Table 2). Similarly, changes in these biomarkers over 6 or 12 months were not significantly associated with relative changes in FVC nor D_LCO_ in any follow-up period (data not shown). In contrast, GAP score at baseline was significantly associated with relative changes in FVC over 12 months (R = − 0.23, *P =* 0.042), and age was significantly associated with relative change in D_LCO_ over 6 and 12 months (R = − 0.26 and − 0.27, *P =* 0.0098 and 0.043).

**Table 2:** Correlation between clinical data at baseline and changes in pulmonary function.

A						
	Relative change in FVC (%)					
	During 6 months			During 12 months		
	R	95%CI	*P* value	R	95%CI	*P* value
Monomeric periostin (ng/mL)	0.015	− 0.21–0.23	0.40	− 0.12	− 0.35–0.11	0.22
Total periostin (ng/mL)	− 0.027	− 0.25–0.19	0.93	0.025	− 0.21–0.26	0.80
LDH (IU/L)	0.028	− 0.19–0.25	0.76	− 0.048	− 0.28–0.19	0.88
KL-6 (IU/mL)	− 0.0087	− 0.23–0.21	0.96	− 0.029	− 0.23–0.21	0.88
SP-D (ng/mL)	− 0.0011	− 0.22–0.22	0.44	− 0.0011	− 0.22–0.22	0.93
Age (years)	− 0.13	− 0.33–0.087	0.40	− 0.21	− 0.42–0.028	0.065
mMRC grade	0.11	− 0.11–0.32	0.80	− 0.13	− 0.35–0.11	0.24
COPD Assessment Test	− 0.16	− 0.36–0.067	0.12	− 0.18	− 0.40–0.053	0.058
GAP score	− 0.070	− 0.28–0.15	0.68	− 0.23	− 0.44–0.0061	0.042*

B						
	Relative change in D_LCO_ (%)					
	During 6 months			During 12 months		
	R	95%CI	*P* value	R	95%CI	*P* value
Monomeric periostin (ng/mL)	− 0.045	− 0.27–0.18	0.95	− 0.070	− 0.31–0.18	0.99
Total periostin (ng/mL)	− 0.15	− 0.36–0.078	0.52	− 0.056	− 0.29–0.19	0.90
LDH (IU/L)	− 0.0035	− 0.23–0.22	0.78	0.10	− 0.14–0.34	0.48
KL-6 (IU/mL)	0.0020	− 0.22–0.23	0.62	0.13	− 0.12–0.36	0.066
SP-D (ng/mL)	− 0.085	− 0.30–0.14	0.58	0.029	− 0.21–0.27	0.91
Age (years)	− 0.26	− 0.46–0.035	0.0098*	− 0.27	− 0.48–0.025	0.043*
mMRC grade	− 0.045	− 0.27–0.18	0.77	− 0.15	− 0.38–0.094	0.64
COPD Assessment Test	− 0.083	− 0.30–0.15	0.48	− 0.23	− 0.44–0.020	0.10
GAP score	− 0.051	− 0.27–0.18	0.36	− 0.057	− 0.30–0.19	0.55

FVC, forced vital capacity; D_LCO_, diffusing capacity of the lung for carbon monoxide; KL-6, Krebs von den Lungen-6; SP-D, surfactant protein D; LDH, lactate dehydrogenase; mMRC, modified Medical Research Council; COPD, chronic obstructive pulmonary disease; GAP, Gender Age Physiology.

**Figure 2:**
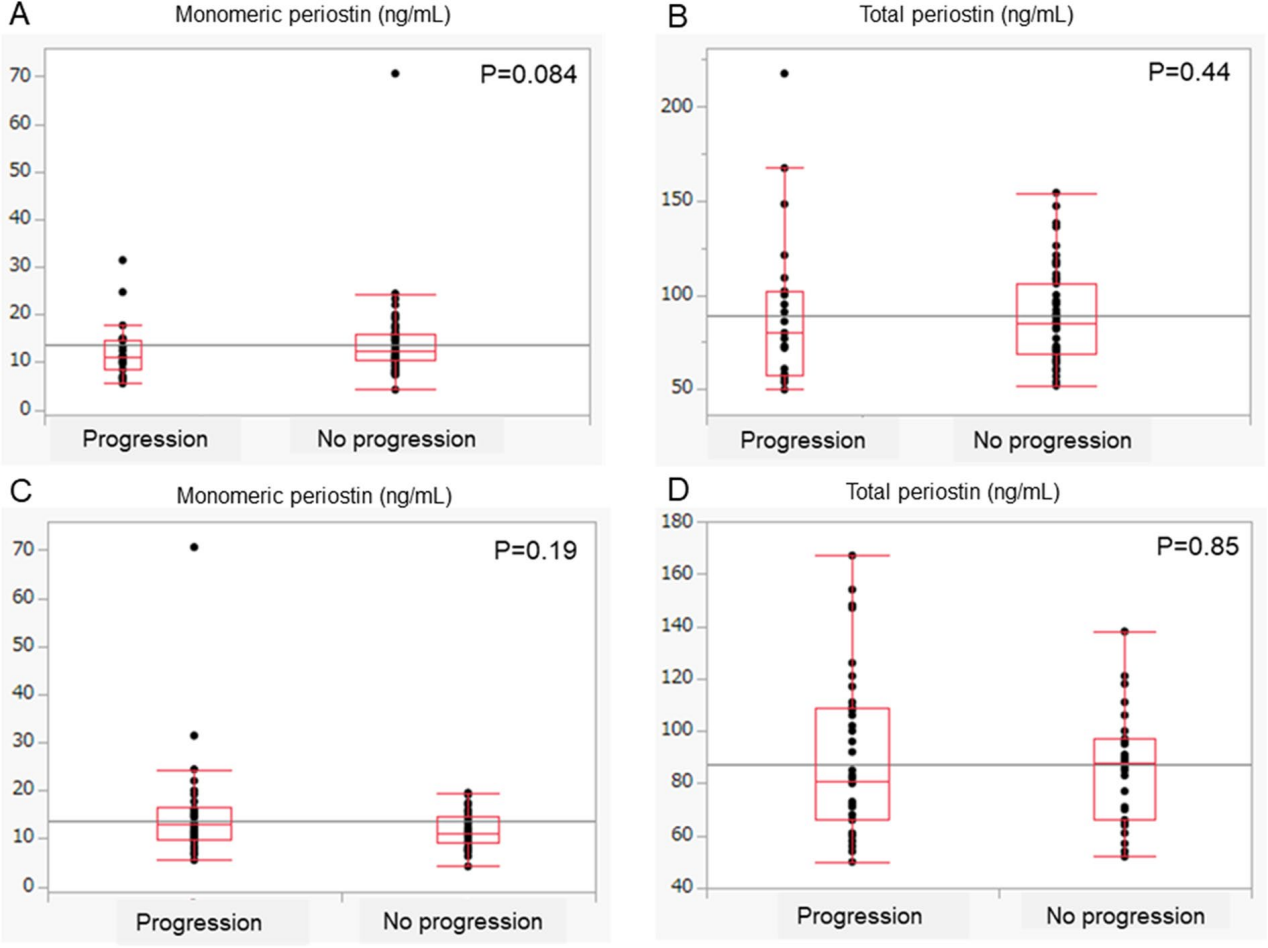
Comparisons of periostin levels between progressive and non-progressive patients after start of nintedanib. Serum monomeric and total periostin levels were compared between progressive and non-progressive participants after start of nintedanib. Monomeric (**A**) and total (**B**) periostin levels were compared between participants with and without a decrease in FVC by more than 5% over a 6 months period. Moreover, monomeric (**C**) and total (**D**) periostin levels were compared between participants with and without a decrease in FVC by more than 5% over a 12 months period. FVC, forced vital capacity. *P < 0.05 was considered to represent statistical significance.

Moreover, we compared serum biomarker levels between progressive and non-progressive patients after the start of treatment with nintedanib. The results of comparing monomeric and total periostin levels between patients with and without a decrease greater than 5% over 6 or 12 months is shown in Fig. 2. Among 80 patients who underwent a pulmonary function test 6 months after the start of nintedanib, 27 (34%) had a greater than 5% decrease in the FVC. Among 71 patients who underwent a pulmonary function test 12 months after the start of nintedanib, 38 (54%) had a greater than 5% decrease in the FVC. There were no significant differences in monomeric and total periostin levels between patients with and without progression in the analyses of 6- and 12-month changes as follows: monomeric periostin, 11.0 (8.4–14.5) ng/mL vs 12.5 (10.3–16.1) ng/mL (*P =* 0.084) and 12.9 (9.9–16.6) ng/mL vs 11.1 (9.2–14.7) ng/mL (*P =* 0.19); total periostin, 80.0 (58.0–102.0) ng/mL vs. 85.0 (69.0–106.0) ng/mL (*P =* 0.44) and 81.0 (66.0–109.0) ng/mL vs. 88.0 (66.0–97.0) ng/mL (*P =* 0.84). Similarly, there were no significant differences in other biomarker levels between patients with and without progression in the analyses of 6- and 12-month changes as follows: LDH, 217.0 (197.0–234.0) IU/L vs. 208.0 (189.5–236.0) IU/L (*P =* 0.51) and 216.5 (193.5–239.3) vs 214.0 (194.0–228.5) IU/L (*P =* 0.61); KL-6, 797.0 (691.0 vs 1286.0) IU/mL vs 855.0 (598.5–1160.0) IU/mL (*P =* 0.58) and 837.0 (611.0–1319.5) IU/mL vs 774.7 (594.0–1149.0) IU/mL (*P =* 0.54); SP-D, 275.0 (222.0 vs. 311.0) IU/mL vs. 272.0 (137.0–415.0) IU/mL (*P =* 0.85) and 273.5 (161.8–352.8) IU/mL vs. 275.0 (127.0–433.0) IU/mL (*P =* 0.88). Neither serum monomeric nor total periostin level was associated with changes in pulmonary function for up to 12 months.

### Analysis of risk factors associated with outcome using Cox proportional hazards model

**Figure 3:**
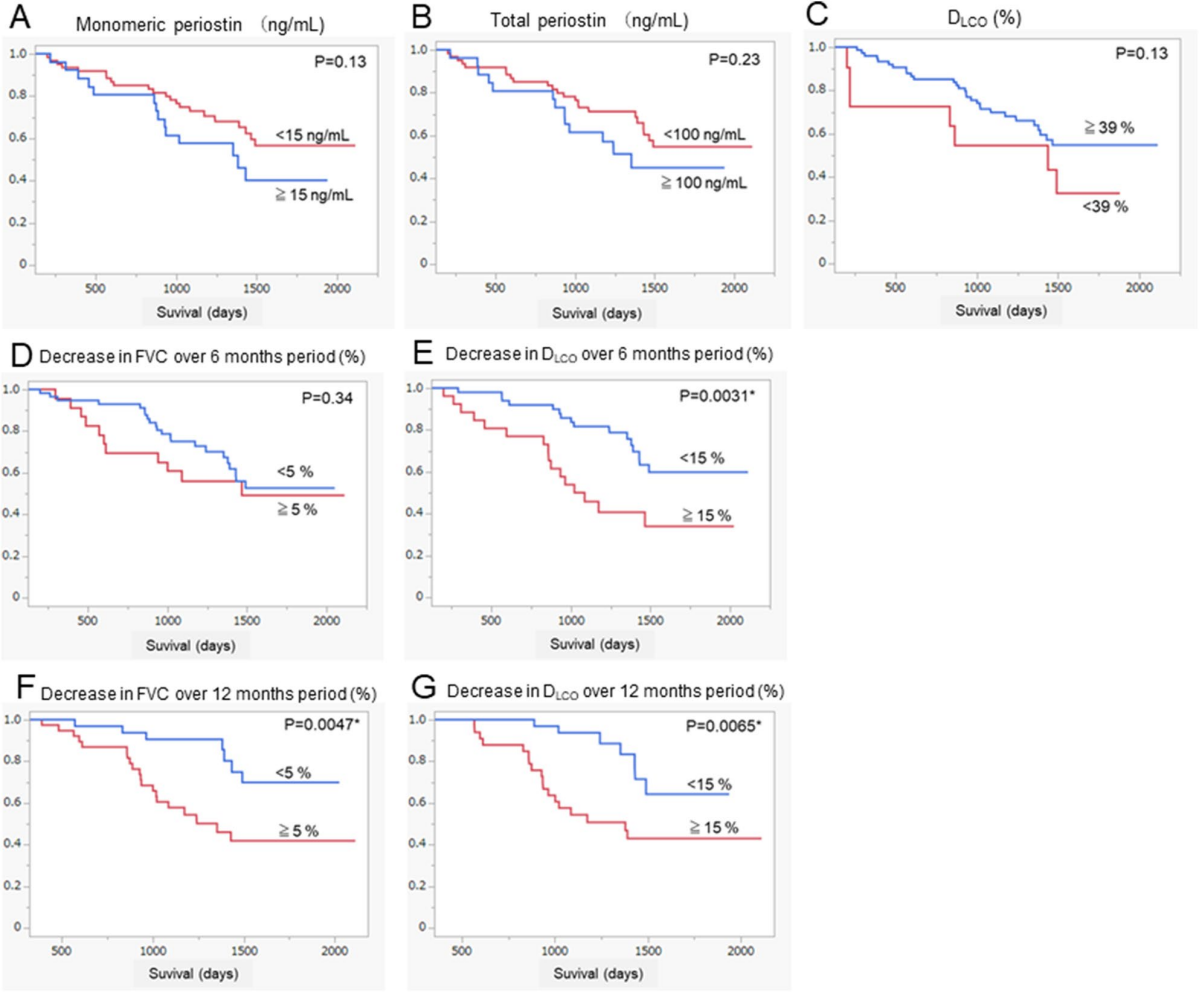
Kaplan–Meier curves for overall survival. Survival curves for overall survival using the Kaplan–Meier Method. Variables significantly associated or tended to be associated with overall survival in univariate analysis of the Cox proportional hazards model including monomeric periostin (**A**), total periostin (**B**), D_LCO_ (**C**), decreases in FVC (**D**) and D_LCO_ (**E**) over 6 months period, and decreases in FVC (**F**) and D_LCO_ (**G**) over 12 months period were analyzed using log-rank test. FVC, forced vital capacity; D_LCO_, diffusing capacity of the lung for carbon monoxide. *P < 0.05 was considered to represent statistical significance.

The results of the Cox proportional hazards model are shown in Table 3. We attempted to clarify whether baseline or changes in serum periostin levels were associated with overall survival. Among clinical data at baseline, higher monomeric (relative risk (RR) 6.1; 95% confidential interval (CI) 1.0–36.0; *P =* 0.047) and total periostin (RR 6.6; 95%CI 1.2–36.3; *P =* 0.031) and lower D_LCO_ (RR 0.012; 95%CI 0.00074–0.19; *P =* 0.0018) were significantly associated with shortened overall survival in the univariate analysis (Table 3). While adjusting for some combinations of confounding factors that are potentially associated with shortened overall survival including age, gender, FVC, and D_LCO_ at baseline in the multivariate analysis, higher monomeric and total periostin levels at baseline remained significant or tended to be risk factors of shortened overall survival (Table 4A,B). In the univariate analysis of changes in clinical data over 6 months, only lower changes in D_LCO_ were significantly associated with shortened overall survival (RR 0.027; 95%CI 0.0032–0.23; *P =* 0.0010), with lower changes in FVC tending to be associated (RR 0.059; 95%CI 0.0035–1.0; *P =* 0.050) (Table 3). Similarly, higher changes in total periostin (RR 57.0; 95%CI 4.0–801.0; *P =* 0.0027) and lower changes in FVC (RR 0.027; 95%CI 0.0029–0.25; *P =* 0.0014) and D_LCO_ (RR 0.014; 95%CI 0.0012–0.15; *P =* 0.00050) over 12 months were significantly associated with shortened overall survival (Table 3). While adjusting for some combinations of changes in FVC and/or D_LCO_, risk factors of mortality in the multivariate analysis, neither change in monomeric and total periostin levels was significantly associated with overall survival (Table 4C,D). Baseline levels and changes in biomarkers other than periostin, including LDH, KL-6, and SP-D, were not associated with overall survival (Table 3). Variables that were significantly associated or tended to be associated with the overall survival in the univariate analysis of the Cox proportional hazards model including monomeric periostin, total periostin, D_LCO_, decrease in FVC and D_LCO_ over 6 months, and decrease in FVC and D_LCO_ over 12 months were analyzed using the Kaplan–Meier method (Fig. 3). Among these variables, a high decrease in the FVC over 12 months (*P =* 0.0031) and D_LCO_ over 6 and 12 months (*P =* 0.0047 and 0.0065) were significantly associated with a shortened overall survival. Moreover, neither serum levels of monomeric nor total periostin, LDH, KL-6, nor SP-D were associated with incidence of acute exacerbation of IPF (data not shown). In the present study, monomeric and total periostin levels at baseline were independent risk factors for mortality but not other biomarkers including LDH, KL-6, and SP-D.

**Table 3:** Univariate analysis of factors associated with overall survival using the Cox proportional hazards model.

A. Baseline data			
	Univariate analysis		
	RR	95%CI	*P* value
Definite UIP pattern on HRCT	2.0	0.49–8.5	0.33
Age	2.2	0.27–18.9	0.46
Gender Male	0.43	0.18–1.04	0.062
Smoker	0.50	0.21–1.2	0.13
GAP score	1.6	0.17–14.9	0.68
More or equal than GAP stage 2	0.85	0.44–1.7	0.64
mMRC grade	1.4	0.78–2.67	0.24
COPD Assessment Test	1.5	0.38–6.0	0.56
Blood data			
Monomeric periostin (ng/mL)	6.1	1.0–36.0	0.047*
Total periostin (ng/mL)	6.6	1.2–36.3	0.031*
LDH(IU/l)	1.3	0.16–9.8	0.82
KL-6 (IU/ml)	2.0	0.33–12.1	0.45
SP-D (ng/mL)	0.42	0.062–2.8	0.37
Pulmonary function test			
FVC (%)	0.41	0.078–2.2	0.30
D_LCO_ (%)	0.012	0.00074–0.19	0.0018*
Six-minutes walk test			
Minimum SpO_2_ (%)	0.23	0.032–1.7	0.15
Distance (m)	0.40	0.086–1.9	0.24
Combination therapy at start of nintedanib			
Corticosteroid	2.2	0.76–6.1	0.15
Long-term oxygen therapy	2.1	0.80–5.3	0.13

B. Changes in clinical data			
	Univariate analysis		
	RR	95%CI	*P* value
Change during 6 months from baseline			
Blood biomarker			
Monomeric periostin (ng/mL)	0.26	0.024–2.8	0.27
Total periostin (ng/mL)	2.5	0.057–112.4	0.63
LDH(IU/l)	1.4	0.049–39.1	0.85
KL-6 (IU/ml)	0.37	0.029–4.9	0.45
SP-D (ng/mL)	1.7	0.29–10.3	0.55
Pulmonary function			
FVC (%)	0.059	0.0035–1.0	0.050
D_LCO_ (%)	0.027	0.0032–0.23	0.0010*
Change during 12 months from baseline			
Blood biomarker			
Monomeric periostin (ng/mL)	3.8	0.46–30.9	0.22
Total periostin (ng/mL)	57.0	4.0–801.0	0.0027*
LDH(IU/l)	4.0	0.22–71.3	0.35
KL-6 (IU/ml)	1.2	0.12–11.7	0.88
SP-D (ng/mL)	0.98	0.17–5.7	0.99
Pulmonary function			
FVC (%)	0.027	0.0029–0.25	0.0014*
D_LCO_ (%)	0.014	0.0012–0.15	0.00050*

RR, relative risk; 95%CI 95% confidential interval; LDH, lactate dehydrogenase; KL-6, Krebs von den Lungen-6; SP-D, surfactant protein D; FVC, forced vital capacity; D_LCO_, diffusing capacity of the lung for carbon monoxide.

**Table 4:** Multivariate analysis of factors associated with overall survival using the Cox proportional hazards model.

A. Monomeric periostin levels and adjusted factors at baseline								
	Model							
	1	2	3	4	5	6	7	8
Monomeric periostin (ng/mL)	6.6 (1.2–36.3)	6.4 (1.1–36.8)	7.4 (1.3–41.9))	7.2 (1.2–42.9)	6.1 (1.1–34.6)	6.0 (0.75–25.6)	5.8 (1.0–32.4)	5.7 (0.86–37.8)
	*P =* 0.031*	*P =* 0.036*	*P =* 0.023*	*P =* 0.031*	*P =* 0.026*	*P =* 0.040*	*P =* 0.045*	*P =* 0.071
Age (years)		2.0 (0.23–17.0)		2.6 (1.1–6.4)				3.8 (0.53–27.5)
		*P =* 0.54		*P =* 0.41				*P =* 0.18
Gender Male			0.41 (0.17–1.0)	0.39 (0.16–0.95)				0.37 (0.15–1.0)
			*P =* 0.050	*P =* 0.039*				*P =* 0.034*
FVC (%)					0.45 (0.082–2.5)		0.64 (0.11–3.7)	0.73 (0.12–4.7)
					*P =* 0.37		*P =* 0.62	*P =* 0.75
D_LCO_ (%)						0.012 (0.00067–0.20)	0.013 (0.00074–0.23)	0.0082 (0.00044–0.15)
						*P =* 0.0022*	*P =* 0.0032*	*P =* 0.0013*

B. Total periostin levels and adjusted factors at baseline								
Variable	Model							
	1	2	3	4	5	6	7	8
Total periostin (ng/mL)	6.1 (1.0–36.0)	5.7 (0.91–35.6)	5.6 (0.99–31.9)	4.9 (0.80–29.9)	6.5 (1.1–38.4)	9.6 (1.8–57.7)	9.5 (1.7–51.4)	6.3 (1.1–35.4)
	*P =* 0.047*	*P =* 0.064	*P =* 0.052	*P =* 0.085	*P =* 0.039*	*P =* 0.0091*	*P =* 0.0092*	*P =* 0.053
Age (years)		1.4 (0.16–12.8)		1.9 (0.19–19.1)				3.1 (0.43–22.5)
		*P =* 0.74		*P =* 0.063				*P =* 0.62
Gender Male			0.44 (0.18–1.1)	0.44 (0.18–1.1)				0.43 (0.17–1.1)
			*P =* 0.073	*P =* 0.58				*P =* 0.092
FVC (%)					0.37 (0.072–1.9)		0.58 (0.10–3.4)	0.67 (0.11–4.2)
					*P =* 0.24		*P =* 0.55	*P =* 0.47
D_LCO_ (%)						0.0081 (0.00050–0.13)	0.0097 (0.00057–0.17)	0.0068 (0.00037–0.12)
						*P =* 0.00070*	*P =* 0.0014*	*P =* 0.0012*

C. Changes in monomeric and total periostin levels and adjusted factors over 6 months								
	Model							
	1	2	3	4	5	6	7	8
Monomeric periostin (ng/mL)	0.26 (0.024–2.8)	0.26 (0.023–2.9)	0.23 (0.021–2.6)	0.22 (0.021–2.3)				
	*P =* 0.27	*P =* 0.27	*P =* 0.23	*P =* 0.21				
Total periostin (ng/mL)					2.5 (0.057–112.4)	5.1 (0.48–53.1)	3.3 (0.24–44.1)	2.2 (0.19–25.6)
					*P =* 0.63	*P =* 0.18	*P =* 0.37	*P =* 0.54
FVC (%)		0.044 (0.0026–0.75)		0.092 (0.0029–3.0)		0.067 (0.0043–1.0)		0.14 (0.0047–4.2)
		*P =* 0.031*		*P =* 0.18		*P =* 0.053*		*P =* 0.26
D_LCO_ (%)			0.023 (0.0025–0.21)	0.025 (0.0024–0.27)			0.077 (0.011–0.55)	0.028 (0.0028–28)
			*P =* 0.00040*	*P =* 0.0023*			*P =* 0.0011*	*P =* 0.0022*

D. Changes in monomeric and total periostin levels and adjusted factors over 12 months								
	Model							
	1	2	3	4	5	6	7	8
Monomeric periostin (ng/mL)	3.8 (0.46–30.9)	0.26 (0.023–2.9)	6.3 (0.98–40.3)	0.22 (0.021–2.3)				
	*P =* 0.22	*P =* 0.27	*P =* 0.053	*P =* 0.21				
Total periosin (ng/mL)					57.0 (4.0–801.0)	10.4 (0.44–246.6)	1.8 (0.054–62.6)	2.0 (0.049–84.1)
					*P =* 0.0027*	*P =* 0.15	*P =* 0.74	*P =* 0.71
FVC (%)		0.044 (0.0026–0.75)		0.092 (0.0029–3.0)		0.025 (0.0027–0.24)		0.016 (0.00056–0.45)
		*P =* 0.031*		*P =* 0.18		*P =* 0.0013*		*P =* 0.015*
D_LCO_ (%)			0.023 (0.0025–0.21)	0.025 (0.0024–0.27)			0.018 (0.0022–0.14)	0.075 (0.0070–0.79)
			*P =* 0.00080*	*P =* 0.0023*			*P =* 0.00010*	*P =* 0.031*

Each of the eight models in Table 4A–D show the effects of covariates on the associations of monomeric and total periostin levels with overall survival. Model 1 in Table 4A–D and model 5 in Table 4C,D have only the group variable; models 2 to 8 in Table 4A–D and models 2 to 4 and 6 to 8 in Table 4C,D progressively add covariates. Relative risks are shown in the top row, 95% confidence intervals in the middle row, and P-values in the bottom row of each cell.

FVC, forced vital capacity; D_LCO_, diffusing capacity of the lung for carbon monoxide; relative risk is shown in the upper row; 95% confidential interval is shown in parentheses in the middle row; *P*-values are shown in the lower row; *A *P*-value < 0.05 represented statistical significance.

#### Analyses of the associations between serum periostin levels and nintedanib effectiveness

**Figure 4:**
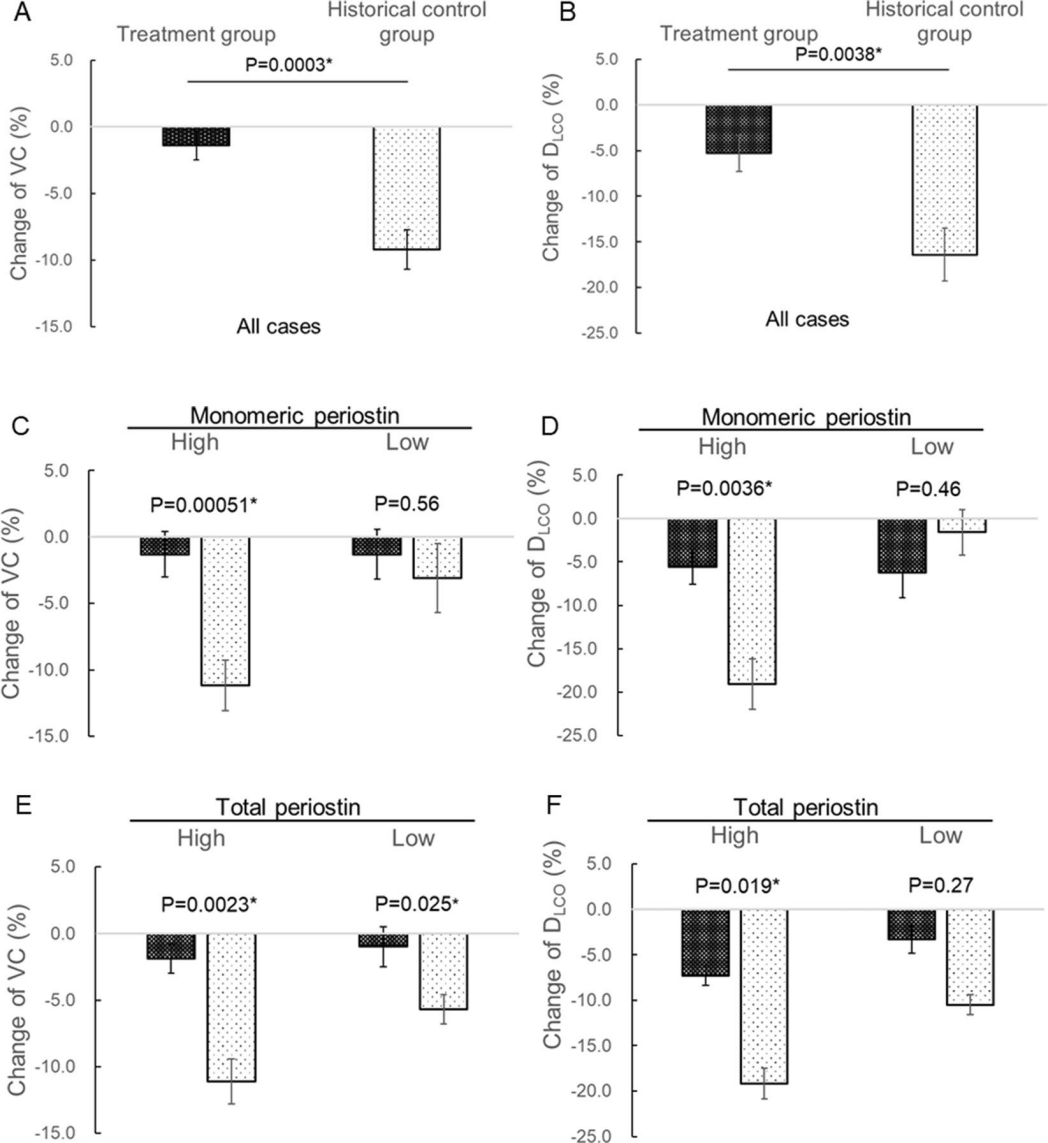

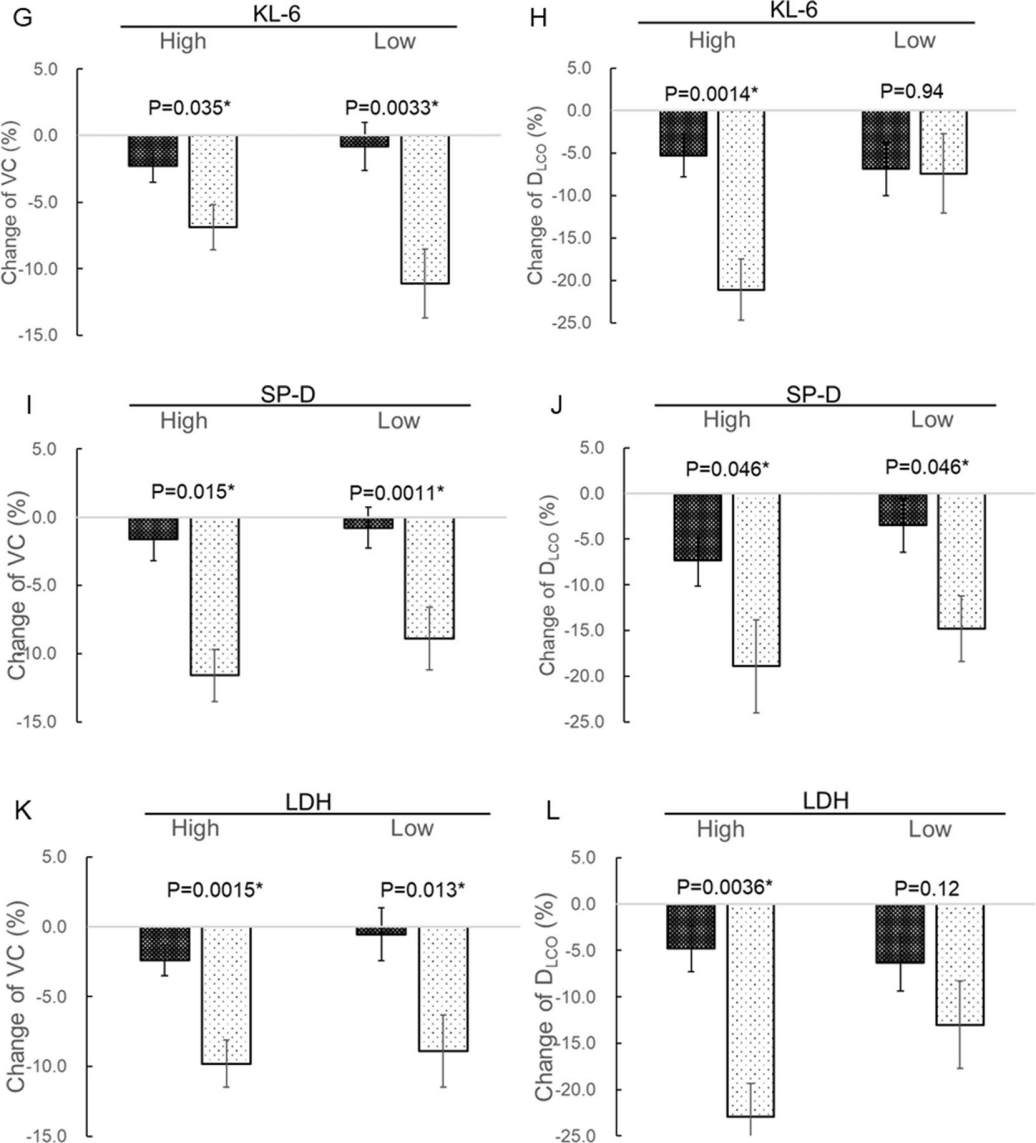
Analysis of the associations between biomarker levels and nintedanib efficacy. As the historical control data, we used serum biomarker levels measured in 43 antifibrotic drug-naive patients with IPF who were selected from 60 patients who participated in previously published studies^28,31^. For evaluating the association between biomarker levels and efficacy of nintedanib, relative changes in VC and D_LCO_ from baseline over 6 months were compared between the study participants (treatment group) and historical controls according to biomarker-high and -low groups. The median of each biomarker in the present study was set as the cut-off level. Statistical analysis for comparing two groups was performed by multiple regression analysis adjusted for age, VC, and D_LCO_ at baseline. Changes in VC, and D_LCO_ in the treatment group are shown by white spots on the black background bar, and those in the historical control group are shown by black spots on the white background bar. Comparisons of VC (**A**) and D_LCO_ (**B**) in all patients in the treatment group and in historical controls are shown. The results of the comparison of decrease in VC are shown in (**C**) (monomeric periostin), (**E**) (total periostin), (**G**) (KL-6), (**I**) (SP-D), and (**K**) (LDH), and those of decrease in D_LCO_ are shown in (**D**) (monomeric periostin), (**F**) (total periostin), (**H**) (KL-6), (**J**) (SP-D), and L (LDH). Data are expressed as the least squares mean ± standard error unless otherwise stated. LDH, lactate dehydrogenase; KL-6, Krebs von den Lungen-6; SP-D, surfactant protein D; VC, vital capacity; D_LCO_, diffusing capacity of the lung for carbon monoxide. *P < 0.05 was considered to represent statistical significance.

A comparison of clinical data at baseline (the time of first measurement of biomarkers) and relative changes in VC and D_LCO_ over 6 months in 80 patients in the present study (treatment group) and 43 in a historical control group (antifibrotic drug-naive patients) are shown in Table 5 and Fig. 4. Seven patients in the treatment group whose pulmonary function at 6 months from baseline could not be measured were excluded. Patients in the treatment group were significantly older and had significantly lower VC than the historical control group (Table 5). There is no significant difference of ILD pattern on HRCT, gender, and rate of smokers and patients treated corticosteroid (Table 5). Decreases in both VC and D_LCO_ in the treatment group were significantly milder than in the historical controls (Fig. 4A,B). A comparison of changes in VC and D_LCO_ according to biomarker-high and -low groups are shown in Fig. 4C–L. In the high monomeric periostin group, decrease in VC in the treatment group was significantly milder than in the historical controls, whereas there was no difference between the two groups in the low expression group (Fig. 4C). In contrast, decrease in VC was significantly milder in the treatment group than in the historical control group, regardless of biomarker levels other than monomeric periostin (Fig. 4E,G,I,K). Among patients with high monomeric and total periostin, KL-6, and LDH, decrease in D_LCO_ in the treatment group was significantly milder than in the historical controls, whereas there was no difference between the two in the low expression group of these biomarkers (Fig. 4D,F,H,L). In contrast, decreased D_LCO_ was significantly milder in the treatment group than in the historical controls, regardless of SP-D level (Fig. 4J). This suggested that some biomarkers were associated with short-term suppressive effects on decrease in pulmonary function following treatment with nintedanib. Among the biomarkers analyzed in the present study, only monomeric periostin was associated with the suppressive effects on decreases in both VC and D_LCO_.

**Table 5:** Comparison of clinical data in patients treated with and without nintedanib.

	Treatment group	Historical controls	*P* value
Number	80	43	
HRCT pattern			
Definite UIP	79 (91%)	35 (81%)	0.16
Possible UIP	8 (9%)	8 (19%)	
Age	72.0 (68.0–76.0)	68.0 (61.0–75.0)	0.014*
Gender Male	78 (90%)	39 (91%)	1.0
Smoker	76 (87%)	36 (84%)	0.60
Pulmonary function test at baseline			
VC (%)	69.8 (60.7–80.1)	86.2 (72.0–101.6)	< 0.0001*
D_LCO_ (%)	57.1 (48.7–67.9)	59.4 (46.4–74.4)	0.59
Treatment with corticosteroid	6 (7%)	2 (5%)	1.0

Data are expressed as the median (25th to 75th percentiles of the interquartile range [IQR]), unless otherwise stated. HRCT, high-resolution computed tomography; UIP, usual interstitial pneumonia; VC, vital capacity; D_LCO_, diffusing capacity of the lung for carbon monoxide.

## Discussion

In the present study, serum monomeric and total periostin were independent risk factors of shortened overall survival but not other biomarkers such as LDH, KL-6, or SP-D in patients treated with nintedanib. Neither monomeric nor total periostin levels were significantly associated with relative changes in FVC or D_LCO_ for up to 1 year. In the analysis of the association between serum biomarker levels and effectiveness of nintedanib therapy, some biomarker levels, particularly those of monomeric periostin, were associated with a suppressive effect on decrease in pulmonary function.

Participants in many previous studies of prognostic factors for ILD, such as GAP stage, were not limited to those treated with antifibrotic drugs^14,15^. In the INMARK trial, a phase 3 RCT of nintedanib therapy for IPF patients with a diagnosis made within the previous 3 years and more than 80% of FVC, the product of C-reactive protein degraded by matrix metalloprotease was associated with changes in FVC for 52 weeks but not the reducing rate of decrease in FVC by nintedanib^17^. The post-hoc analysis of a phase 3 RCT of pirfenidone therapy (ASCEND and CAPACITY 1 and 2 trials) for IPF suggested that evaluations of a combination of CCL18, C-X-C motif chemokine ligand 14, and total periostin were better at predicting the prognosis and therapeutic effect compared with any single biomarker^21^. Adegunsoye et al. performed a pooled, multicenter, propensity-matched analysis of IPF patients with and without antifibrotic drug exposure. In this study, CA-125, CXCL13, MMP7, chitinase-3-like protein-1, and osteopontin predicted differential transplant-free survival in antifibrotic drug-exposed patients but at higher thresholds than in antifibrotic drug-non-exposed individuals^37^. Yoshikawa et al. showed that increased serum levels of SP-A was an independent predictor of disease progression in 43 IPF patients who were treated with antifibrotic drugs^38^.

Decrease in pulmonary function is the most important prognostic factor and the endpoint of prognostic biomarker studies^39^. The result showing no significant association between periostin levels and decrease in pulmonary function in the present study was inconsistent with those in previous studies performed by our and other study groups^30,31,33^. In these previous studies, many study participants were not treated with antifibrotic drugs, whereas all present study participants were treated with nintedanib. The lack of association between periostin levels and decrease in pulmonary function may have been caused by suppressing effect by nintedanib therapy. To resolve this issue, association between serum periostin levels and changes in pulmonary function over 1 year from baseline should be analyzed in a further study.

Treatment with antifibrotic drugs can cause some adverse events such as gastrointestinal involvement, resulting in high medical expenses^9,10,12^. Thus, one of the significant clinical questions of treatment for IPF is whether early intervention with antifibrotic drug therapy for patients with mild disease is appropriate^40,41^. A quantitative, point-in-time data surveillance study of IPF showed that antifibrotic drug use was 60% for all patients, compared with only 37% for those with milder disease with FVC > 75% in Japan at 2019^40^. The results of the present study suggest that early intervention with nintedanib therapy is recommended for patients with high serum periostin levels because they have high mortality and therapeutic responses to nintedanib. Precision medicine in IPF clinical practice remains an unmet need when using biomarkers to predict prognosis and therapeutic effectiveness. Because tailored therapies based on precision medicine can improve treatment outcomes by the early intervention for patients who can benefit from treatment, the development of new biomarkers is needed^42^. Establishment of such biomarkers predicting outcomes of IPF could contribute to decision-making in therapeutic management.

The present study has some limitations. First, the sample size was small. We will plan further studies in larger populations. Second, in the analysis of the association between biomarker levels and changes in pulmonary function, the presence of cases in which pulmonary function could not be measured over time because of worsening conditions should be considered as omitted variable bias. Third, the analysis of associations between biomarker levels and therapeutic effectiveness was limited to comparisons with historical controls. The use of historical controls might cause selection and/or temporal bias due to the patient characteristics or standard of care. However, we think that the results in the present study are reliable because we performed multiple regression analysis using the treatment group and historical controls, adjusting for the VC, D_LCO_, and age. Fourth, in the present study, we set the endpoint as FVC for analyzing the association between biomarker levels and outcome, and VC for comparisons between the treatment group and historical controls, because the endpoint was VC but not FVC in our previous study.

## Conclusions

We showed that serum levels of monomeric and total periostin but not other biomarkers were associated with overall survival in IPF patients who received nintedanib. Moreover, some biomarkers, particularly monomeric periostin, were associated with a suppressive effect on decrease in pulmonary function by nintedanib. Periostin assessments may contribute to determining therapeutic strategies for patients with IPF.

## Data Availability

The datasets used and/or analyzed during the current study available from the corresponding author on reasonable request.

## References

[1] Raghu G, Remy-Jardin M, Richeldi L, Thomson CC, Inoue Y, Johkoh T (2022). Idiopathic pulmonary fibrosis (an Update) and progressive pulmonary fibrosis in adults: An Official ATS/ERS/JRS/ALAT clinical practice guideline. Am. J. Respir. Crit. Care Med..

[2] Raghu G, Remy-Jardin M, Myers JL, Richeldi L, Ryerson CJ, Lederer DJ (2018). Diagnosis of idiopathic pulmonary fibrosis. An official ATS/ERS/JRS/ALAT clinical practice guideline. Am. J. Respir. Crit. Care Med..

[3] Raghu G, Collard HR, Egan JJ, Martinez FJ, Behr J, Brown KK (2011). An official ATS/ERS/JRS/ALAT statement: Idiopathic pulmonary fibrosis: Evidence-based guidelines for diagnosis and management. Am. J. Respir. Crit. Care Med..

[4] Natsuizaka M, Chiba H, Kuronuma K, Otsuka M, Kudo K, Mori M (2014). Epidemiologic survey of Japanese patients with idiopathic pulmonary fibrosis and investigation of ethnic differences. Am. J. Respir. Crit. Care Med..

[5] Flaherty KR, Mumford JA, Murray S, Kazerooni EA, Gross BH, Colby TV (2003). Prognostic implications of physiologic and radiographic changes in idiopathic interstitial pneumonia. Am. J. Respir. Crit. Care Med..

[6] Zappala CJ, Latsi PI, Nicholson AG, Colby TV, Cramer D, Renzoni EA (2010). Marginal decline in forced vital capacity is associated with a poor outcome in idiopathic pulmonary fibrosis. Eur. Respir. J..

[7] Latsi PI, du Bois RM, Nicholson AG, Colby TV, Bisirtzoglou D, Nikolakopoulou A (2003). Fibrotic idiopathic interstitial pneumonia: The prognostic value of longitudinal functional trends. Am. J. Respir. Crit. Care Med..

[8] Mogulkoc N, Brutsche MH, Bishop PW, Greaves SM, Horrocks AW (2001). Greater Manchester Pulmonary Fibrosis Consortium. Pulmonary function in idiopathic pulmonary fibrosis and referral for lung transplantation. Am. J. Respir. Crit. Care Med..

[9] Richeldi L, du Bois RM, Raghu G, Azuma A, Brown KK, Costabel U (2014). Efficacy and safety of nintedanib in idiopathic pulmonary fibrosis. N. Engl. J. Med..

[10] King TE, Bradford WZ, Castro-Bernardini S, Fagan EA, Glaspole I, Glassberg MK (2014). A phase 3 trial of pirfenidone in patients with idiopathic pulmonary fibrosis. N. Engl. J. Med..

[11] Petnak T, Lertjitbanjong P, Thongprayoon C, Moua T (2021). Impact of antifibrotic therapy on mortality and acute exacerbation in idiopathic pulmonary fibrosis: A systematic review and meta-analysis. Chest.

[12] Homma S, Bando M, Azuma A, Sakamoto S, Sugino K, Ishii Y (2018). Japanese guideline for the treatment of idiopathic pulmonary fibrosis. Respir Investig..

[13] Ley B, Bradford WZ, Vittinghoff E, Weycker D, du Bois RM, Collard HR (2016). Predictors of mortality poorly predict common measures of disease progression in idiopathic pulmonary fibrosis. Am. J. Respir. Crit. Care Med..

[14] Ryerson CJ, Vittinghoff E, Ley B, Lee JS, Mooney JJ, Jones KD (2014). Predicting survival across chronic interstitial lung disease: The ILD-GAP model. Chest.

[15] Kondoh S, Chiba H, Nishikiori H, Umeda Y, Kuronuma K, Otsuka M (2016). Validation of the Japanese disease severity classification and the GAP model in Japanese patients with idiopathic pulmonary fibrosis. Respir. Investig..

[16] Kreuter M, Lee JS, Tzouvelekis A, Oldham JM, Molyneaux PL, Weycker D (2021). Monocyte count as a prognostic biomarker in patients with idiopathic pulmonary fibrosis. Am. J. Respir. Crit. Care Med..

[17] Maher TM, Stowasser S, Nishioka Y, White ES, Cottin V, Noth I (2019). Biomarkers of extracellular matrix turnover in patients with idiopathic pulmonary fibrosis given nintedanib (INMARK study): A randomised, placebo-controlled study. Lancet Respir. Med..

[18] Organ LA, Duggan AR, Oballa E, Taggart SC, Simpson JK, Kang'ombe AR (2019). Biomarkers of collagen synthesis predict progression in the PROFILE idiopathic pulmonary fibrosis cohort. Respir. Res..

[19] Rosas IO, Richards TJ, Konishi K, Zhang Y, Gibson K, Lokshin AE (2008). MMP1 and MMP7 as potential peripheral blood biomarkers in idiopathic pulmonary fibrosis. PLoS Med..

[20] Prasse A, Probst C, Bargagli E, Zissel G, Toews GB, Flaherty KR (2009). Serum CC-chemokine ligand 18 concentration predicts outcome in idiopathic pulmonary fibrosis. Am. J. Respir. Crit. Care Med..

[21] Neighbors M, Cabanski CR, Ramalingam TR, Sheng XR, Tew GW, Gu C (2018). Prognostic and predictive biomarkers for patients with idiopathic pulmonary fibrosis treated with pirfenidone: Post-hoc assessment of the CAPACITY and ASCEND trials. Lancet Respir. Med..

[22] Yokoyama A, Kondo K, Nakajima M, Matsushima T, Takahashi T, Nishimura M (2006). Prognostic value of circulating KL-6 in idiopathic pulmonary fibrosis. Respirology.

[23] Takahashi H, Fujishima T, Koba H, Murakami S, Kurokawa K, Shibuya Y (2000). Serum surfactant proteins A and D as prognostic factors in idiopathic pulmonary fibrosis and their relationship to disease extent. Am. J. Respir. Crit. Care Med..

[24] Ishikawa N, Hattori N, Yokoyama A, Kohno N (2012). Utility of KL-6/MUC1 in the clinical management of interstitial lung diseases. Respir. Investig..

[25] Conway SJ, Izuhara K, Kudo Y, Litvin J, Markwald R, Ouyang G (2014). The role of periostin in tissue remodeling across health and disease. Cell Mol. Life Sci..

[26] Yamaguchi Y, Ono J, Masuoka M, Ohta S, Izuhara K, Ikezawa Z (2013). Serum periostin levels are correlated with progressive skin sclerosis in patients with systemic sclerosis. Br. J. Dermatol..

[27] Takayama G, Arima K, Kanaji T, Toda S, Tanaka H, Shoji S (2006). Periostin: A novel component of subepithelial fibrosis of bronchial asthma downstream of IL-4 and IL-13 signals. J. Allergy Clin. Immunol..

[28] Okamoto M, Izuhara K, Ohta S, Ono J, Hoshino T (2019). Ability of periostin as a new biomarker of idiopathic pulmonary fibrosis. Adv. Exp. Med. Biol..

[29] Uchida M, Shiraishi H, Ohta S, Arima K, Taniguchi K, Suzuki S (2012). Periostin, a matricellular protein, plays a role in the induction of chemokines in pulmonary fibrosis. Am. J. Respir. Cell Mol. Biol..

[30] Okamoto M, Hoshino T, Kitasato Y, Sakazaki Y, Kawayama T, Fujimoto K (2011). Periostin, a matrix protein, is a novel biomarker for idiopathic interstitial pneumonias. Eur. Respir. J..

[31] Naik PK, Bozyk PD, Bentley JK, Popova AP, Birch CM, Wilke CA (2012). Periostin promotes fibrosis and predicts progression in patients with idiopathic pulmonary fibrosis. Am. J. Physiol .Lung Cell Mol. Physiol..

[32] Tajiri M, Okamoto M, Fujimoto K, Johkoh T, Ono J, Tominaga M (2015). Serum level of periostin can predict long-term outcome of idiopathic pulmonary fibrosis. Respir. Investig..

[33] Ohta S, Okamoto M, Fujimoto K, Sakamoto N, Takahashi K, Yamamoto H (2017). The usefulness of monomeric periostin as a biomarker for idiopathic pulmonary fibrosis. PLoS ONE.

[34] Shimizu H, Sakamoto S, Okamoto M, Isshiki T, Ono J, Shimizu S (2021). Association of serum monomeric periostin level with outcomes of acute exacerbation of idiopathic pulmonary fibrosis and fibrosing nonspecific interstitial pneumonia. Ann. Transl. Med..

[35] Nukui Y, Miyazaki Y, Masuo M, Okamoto T, Furusawa H, Tateishi T (2019). Periostin as a predictor of prognosis in chronic bird-related hypersensitivity pneumonitis. Allergol. Int..

[36] Collard HR, Moore BB, Flaherty KR, Brown KK, Kaner RJ, King TE (2007). Acute exacerbations of idiopathic pulmonary fibrosis. Am. J. Respir. Crit. Care Med..

[37] Adegunsoye A, Alqalyoobi S, Linderholm A, Bowman WS, Lee CT, Pugashetti JV (2020). Circulating plasma biomarkers of survival in antifibrotic-treated patients with idiopathic pulmonary fibrosis. Chest.

[38] Yoshikawa T, Otsuka M, Chiba H, Ikeda K, Mori Y, Umeda Y (2020). Surfactant protein A as a biomarker of outcomes of anti-fibrotic drug therapy in patients with idiopathic pulmonary fibrosis. BMC Pulm Med..

[39] Inoue Y, Kaner RJ, Guiot J, Maher TM, Tomassetti S, Moiseev S (2020). Diagnostic and prognostic biomarkers for chronic fibrosing interstitial lung diseases with a progressive phenotype. Chest.

[40] Lancaster L, Bonella F, Inoue Y, Cottin V, Siddall J, Small M (2022). Idiopathic pulmonary fibrosis: Physician and patient perspectives on the pathway to care from symptom recognition to diagnosis and disease burden. Respirology.

[41] Salisbury ML, Conoscenti CS, Culver DA, Yow E, Neely ML, Bender S (2020). Antifibrotic drug use in patients with idiopathic pulmonary fibrosis. Data from the IPF-PRO registry. Ann. Am. Thorac. Soc..

[42] Karampitsakos T, Juan-Guardela BM, Tzouvelekis A, Herazo-Maya JD (2023). Precision medicine advances in idiopathic pulmonary fibrosis. EBioMedicine..

